# Heterobimetallic Zeolite, InV-ZSM-5, Enables Efficient Conversion of Biomass Derived Ethanol to Renewable Hydrocarbons

**DOI:** 10.1038/srep16039

**Published:** 2015-11-03

**Authors:** Chaitanya K. Narula, Zhenglong Li, Erik M. Casbeer, Robert A. Geiger, Melanie Moses-Debusk, Martin Keller, Michelle V. Buchanan, Brian H. Davison

**Affiliations:** 1 Materials Science & Technology Division, Oak Ridge National Laboratory, Oak Ridge, TN, USA, 37831-6133; 2 Energy & Environmental Sciences Directorate, Oak Ridge National Laboratory, Oak Ridge, TN 37831; 3 Physical Sciences Directorate, Oak Ridge National Laboratory, Oak Ridge, TN 37831.

## Abstract

Direct catalytic conversion of ethanol to hydrocarbon blend-stock can increase biofuels use in current vehicles beyond the ethanol blend-wall of 10–15%. Literature reports describe quantitative conversion of ethanol over zeolite catalysts but high C_2_ hydrocarbon formation renders this approach unsuitable for commercialization. Furthermore, the prior mechanistic studies suggested that ethanol conversion involves endothermic dehydration step. Here, we report the complete conversion of ethanol to hydrocarbons over InV-ZSM-5 without added hydrogen and which produces lower C_2_ (<13%) as compared to that over H-ZSM-5. Experiments with C_2_H_5_OD and *in situ* DRIFT suggest that most of the products come from the hydrocarbon pool type mechanism and dehydration step is not necessary. Thus, our method of direct conversion of ethanol offers a pathway to produce suitable hydrocarbon blend-stock that may be blended at a refinery to produce fuels such as gasoline, diesel, JP-8, and jet fuel, or produce commodity chemicals such as BTX.

The expansion of biomass derived ethanol industry and concurrent development of distribution channels during the last 20 years have created interest in ethanol conversion to industrial chemicals. The commercialization of ethylene derived from bioethanol represents a successful deployment of a renewable industrial chemical^1^,^2^. The addition of biomass derived ethanol to gasoline in the transportation sector is an important step in the utilization of renewable energy^3^. The energy independence and security act of 2007 requires 36 billion gallons of biomass derived fuel by 2022 but ethanol demand is capped at ~14 billion gallons due to “blend-wall” at 10–15%. Although a higher concentration of ethanol containing fuel (e.g., E85) has been available for several years, such fuel can be used only in flex-fuel vehicles whose U.S. market penetration is low^4^. This has renewed interest in the conversion of ethanol to hydrocarbon blend-stock and other industrial chemicals.

Ethanol conversion to hydrocarbons employing zeolites as catalysts dates back to 1970s^5^. Since then, a large number of reports have appeared in literature on ethanol conversion to hydrocarbons^6^,^7^,^8^,^9^,^10^,^11^,^12^,^13^,^14^,^15^,^16^,^17^,^18^,^19^,^20^,^21^,^22^,^23^,^24^,^25^,^26^,^27^,^28^,^29^,^30^,^31^,^32^,^33^,^34^,^35^,^36^,^37^,^38^,^39^. The reaction temperature for ethanol transformation is generally >350 °C and the pressure ranges from ambient to several atmospheres^6^,^7^,^8^,^9^,^10^,^11^,^12^,^13^,^14^,^15^,^16^,^17^,^18^,^19^,^20^,^21^,^22^,^23^,^24^,^25^,^26^,^27^,^28^,^29^,^30^,^31^,^32^,^33^,^34^,^35^,^36^,^37^,^38^,^39^. The product stream is generally high in C_2_ hydrocarbons (e.g., ethylene and ethane), which are not valuable for liquid fuel production or commodity chemical production (separation of pure ethylene is quite expensive from a mixed stream). The mechanism of ethanol conversion is still being debated. A simple mechanism with ethanol dehydration to ethylene or diethyl ether as the first step and subsequent upgrading to C_3+_ hydrocarbons was proposed in 1978 by Derouane *et al.*^5^ and reaffirmed in 2006 by Inaba *et al.*^40^. Recently, a hydrocarbon pool pathway for ethanol conversion has also been proposed in analogy with methanol conversion process^41^. A comprehensive study of decayed catalyst due to extensive coking clearly shows the presence of free radicals and their role in the ethanol transformation is being studied by Madiera *et al.* and Pinard *et al.*^42^,^43^,^44^. A recent review article summarizes state of the understanding of ethanol conversion mechanism over zeolites stating that ethanol first dehydrates to ethylene which then undergoes oligomerization to produce various olefins, paraffins, cyclics, and aromatics^45^. In addition, radicals provide additional active sites for secondary reactions of ethylene^45^. Despite these advances, the technology did not go beyond laboratory until recently^46^ due to lack of information on optimized conversion conditions, catalyst durability, and a clear understanding of mechanism to determine energy balance of ethanol conversion.

Here, we report a versatile heterobimetallic catalyst, InV-ZSM-5, that completely converts ethanol to hydrocarbons in 250–450 °C range and atmospheric pressure without added hydrogen. The heterometallic zeolites, MM’-ZSM-5, are a new class of zeolites where the interaction between M and M’ plays an important role^47^. The InV-ZSM-5 catalyst exhibits superior performance compared to monometallic catalysts, In-ZSM-5 or V-ZSM-5 as determined by low C_2_ yield and high durability. All these catalysts are robust to water content in ethanol (5–95%) and volatile impurities in fermentation stream. Furthermore, our experiments with deuterium labeling and *in situ* DRIFTS experiments rule out ethanol dehydration as a necessary step in ethanol upgrading.

## Results and Discussion

### Catalyst synthesis and characterization

The samples of InV-ZSM-5 were synthesized by ion exchange methods. For comparison, we also prepared samples of H-ZSM-5, V-ZSM-5 and In-ZSM-5. Among these zeolites, H-ZSM-5 is the most extensively explored zeolite^45^. We chose V-ZSM-5 because it has been shown to be highly effective oxydehydrogenation catalyst^48^. For V-ZSM-5, Somorjai *et al.* pointed out that vanadium exchange leads to 95% removal of the Brønsted sites^48^. We expected inhibition of ethanol dehydration over V-ZSM-5 as compared with H-ZSM-5 resulting in reduced ethylene (C_2_) production. In order to increase oligomerization and aromatization, we modified V-ZSM-5 with In since In-ZSM-5 is a well-known alkane aromatization catalyst^49^. The In-ZSM-5 was also investigated to ensure that performance of heterobimetallic zeolite, InV-ZSM-5, is not a sum of V-ZSM-5 and In-ZSM-5. Characterization of InV-ZSM-5 is quite challenging. We have previously shown that characterization tools such as X-ray diffraction, electron microscopy, X-ray absorption near edge spectroscopy, etc., are not helpful in discerning the structure of heterobimetallic zeolites^47^. Hutchings *et al.* have also extensively explored the structure of bimetallic zeolites by a variety of methods^50^. We found that the most useful information on heterobimetallic zeolites, MM’-ZSM-5, is obtained from elemental analysis to show that both M and M’ are present in MM’-ZSM-5 and UV-Vis to ascertain the presence of small clusters of M’. Finally, the reactivity is an excellent indication of the behavior of bimetallic zeolites. In our previous work, we found the activity of heterobimetallic zeolite, CuFe-ZSM-5, to be better than individual Cu-ZSM-5 or Fe-ZSM-5^47^. The C_2_ and C_3+_ selectivity of InV-ZSM-5 was superior to monometallic V-ZSM-5 or In-ZSM-5 and is described in detail in succeeding section.

**Figure 1:**
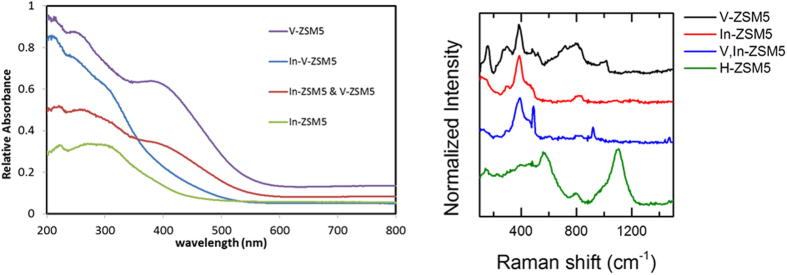
UV-Vis (left) and Raman Spectra (right) of InV-ZSM-5. For comparison, spectra of V-ZSM-5, In-ZSM-5, mechanical mixture of In-ZSM-5 and V-ZSM-5, and H-ZSM-5 are also presented.

A recent report suggests that Raman spectroscopy can be useful in the characterization of heterobimetallic zeolites^51^. Bimetallic InV-ZSM-5 used in this research has 0.6 wt% vanadium and 1.6 wt% indium, indicating both In and V are present. The UV-Vis of InV-ZSM-5 shows an absorption near 300 nm which can originate from In^3+^ and V^5+^ species since both In-ZSM-5 and V-ZSM-5 show absorption near 300 nm (Fig. 1). Furthermore, there is no band at 402 nm which is present in V-ZSM-5. In contrast, the mechanical mixture (V-ZSM-5 and In-ZSM-5 with V:In identical to that of InV-ZSM-5) shows bands at ~300 and ~400 nm. This suggests that our sample of InV-ZSM-5 is not a mechanical mixture of V-ZSM-5 and In-ZSM-5. The UV-Vis data (Fig. 1) on V-ZSM-5 match with literature data and exhibit vanadium bands at 286 and 402 nm which have been previously assigned to Td and Oh V^5+^ 52. As reported previously, the ion exchange leads to monoionic hydroxo- or oxo-indium species at cation sites, the 300 nm absorption in UV-Vis of In-ZSM-5 can be assigned to indium species^53^.

The Raman spectra of our V-ZSM-5 sample (Fig. 1) matches with previously published Raman of V-ZSM-5 prepared from V_2_O_5_ and H-ZSM-5^54^. The In-ZSM-5 shows strongest band at 395 cm^−1^ with shoulders at 350 and 450 cm^−1^. In comparison, cubic In(OH)_3_ exhibits bands at 137, 204, 307, 356, 390, and 659 cm^−1^ 55. After crystallization to In_2_O_3_ at 400 °C, bands at 125, 295, 488, and 615 cm^−1^ are observed^55^. These data show that the indium species in In-ZSM-5 is not surface deposited In_2_O_3_ or In(OH)_3_. The Raman spectrum of a mechanical mixture is identical to that of V-ZSM-5 (not shown in Fig. 1). The Raman spectrum of InV-ZSM-5, on the other hand, is very similar to that of In-ZSM-5. In addition, two sharp peaks are observed at 484 and 919 cm^−1^. These peaks do not match with V_2_O_5_, In_2_O_3_, or VInO_4_. We initially considered the possibility of these peaks due to surface adsorbed species but pretreatment involving heating to 500 °C and cooling to 200 °C in air did not change the spectrum ruling out surface adsorbed species. The additional peaks might be due to complex vanadium and indium interactions.

### Ethanol Conversion to Hydrocarbon Blend-Stock

**Figure 2:**
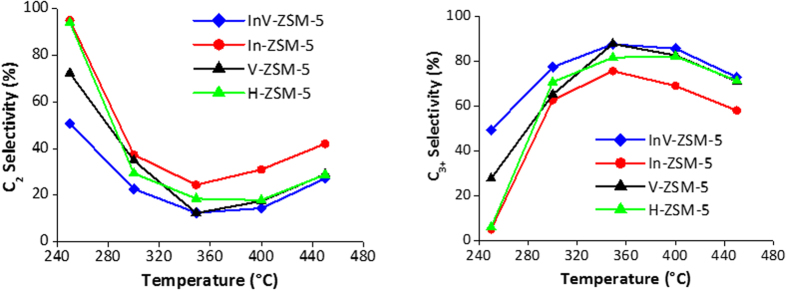
C_2_ selectivity (left) and C_3+_ selectivity (right) as a function of temperature for InV-ZSM-5, V-ZSM-5, In-ZSM-5 and H-ZSM-5 at WHSV of 1.6 h^−1^.

Ethanol conversion to hydrocarbon for all the catalysts studied is 100% in 250 °C to 450 °C range and no oxygenates are observed as shown in Fig. S1. All selectivities shown in Fig. 2 are for 100% ethanol conversion to hydrocarbons after excluding water which is 39.1% of the product stream. The InV-ZSM-5 produces much less C_2_ than V-ZSM-5 in 250–350 °C temperature range (Fig. 2, left), however, there is no difference in C_2_ selectivity for InV-ZSM-5 and V-ZSM-5 in 350–450 °C range. Concurrently, InV-ZSM-5 produces more valuable C_3+_ in higher yields as compared to V-ZSM-5 in 250–350 °C and to In-ZSM-5 in 250–450 °C range. In contrast, the C_2_ selectivity for H-ZSM-5 is higher and C_3+_ is lower as compared to product stream from InV-ZSM-5 in 250–400 °C temperature range (Fig. 2). Thus, lower C_2_ selectivity and higher C_3+_ selectivity of InV-ZSM-5 over V-ZSM-5 or In-ZSM-5 in the desired temperature range makes it more desirable catalyst than monometallic ones or the un-exchanged H-ZSM-5.

The ethanol conversion reaction was run on InV-ZSM-5 after cooling the catalyst from the previous run in 250–450 °C range and the catalyst performance did not change in 250–450 °C range. This suggests that InV-ZSM-5 does not change as a result of exposure to operating conditions in 250–450 °C range. The product stream from ethanol conversion over InV-ZSM-5 contains 19% C_3_-C_4_ olefins, 35% C_3_-C_4_ paraffin and 33% C_5+_ liquid fraction. The C_5+_ liquid fraction has an average molecular weight of 97.6, average specific gravity of 0.81, and Reid Vapor Pressure of 5.14. The calculated research and motor octane numbers are 105.7 and 90.6, respectively. The liquid product stream contains over 350 compounds, consisting of 3.8% paraffins, 24.0% iso-paraffins, 6.5% olefins, 5.4% naphthenes, 60.2% aromatics, and 4.2% unidentified by volume.

### Catalyst Durability

In view of their low C_2_ and high C_3+_ selectivities, the durability studies were limited to InV-ZSM-5 and V-ZSM-5 only. Gas chromatograms of hydrocarbon product streams (Fig. S2), employing 10–100% aqueous ethanol as the input stream, show that the product stream is unaffected by the fraction of water in aqueous ethanol. Regardless of the water fraction in the input stream, the ratios of individual peaks in gas chromatograms remain unchanged under our reaction conditions. Our results are different from previously reported results where water appeared to impact aromatic selectivity^56^. Our results suggest that fermentation streams at any stage of purification can be employed for conversion to hydrocarbon blend-stock because fermentation stream is essentially ethanol and water with small amounts of acetaldehyde, methanol, diacetyl, ethyl acetate, and acetic acid (0.1% total) even after first flash distillation. The output stream contains C_2+_ hydrocarbons and water which can be easily separated. A 40% ethanol solution which also contains volatile fermentation stream impurities of acetaldehyde, methanol, diacetyl, ethyl acetate, and acetic acid (0.1% total) was successfully run for 100 h over V-ZSM-5 (WHSV 1.6 h^−1^) with periodic decoking at 450 °C. The impurities did not impact catalyst or product stream. Thus, partially concentrated ethanol stream can be used for upgrading reaction and expensive complete dewatering is not necessary.

**Figure 3:**
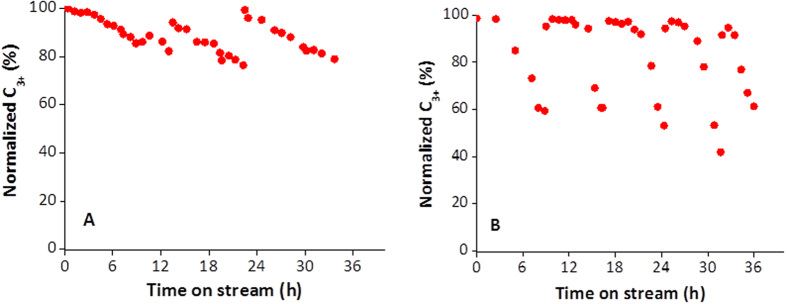
Durability tests for (**left**) InV-ZSM-5 and (**right**) V-ZSM-5 at 350 °C and WHSV of 4.0 h^−1^.

The durability tests for InV-ZSM-5 and V-ZSM-5 were carried out at a WHSV of 4.0 h^−1^ at 350 °C employing pure ethanol (Fig. 3). The catalyst performance was monitored by the yield of C_3+_ hydrocarbons as a function of time (normalized by the C_3+_ yield at the beginning). The C_3+_ yield is shown as 100% when catalyst is fresh and drops to ~80%. InV-ZSM-5 was on stream for about 12 h before C_3+_ yield dropped below 80%. The loss in performance is due to coking, which is commonly observed in alcohol or hydrocarbon upgrading on zeolites. After decoking at 450 °C, the catalyst only recovers about 94% activity because the temperature is not high enough for complete decoking to fully recover its performance. After decoking at 500 °C, the catalyst fully recovers its performance. In contrast, the coking of V-ZSM-5 occurs at about 6 h at WHSV of 4.0 h^−1^. After decoking, the catalyst regains its performance.

Overall, InV-ZSM-5 can function twice as long as V-ZSM-5 before requiring decoking. The enhanced durability can be assigned to the incorporation of In in InV-ZSM-5. Thus, the higher C_3+_ selectivity, described in the preceding section, and the enhanced durability of InV-ZSM-5 make it a more desirable catalyst than V-ZSM-5.

### Mechanism

As discussed in the introduction section, there are three different proposed mechanisms for ethanol conversion to hydrocarbons – dehydration, hydrocarbon pool, and free radical. For all three pathways, the ethanol dehydration is considered to be first step. In our work, we found that ethanol conversion to hydrocarbons is exothermic (ΔHr, −1.0 MJ/kg) based on heat of formation data. Here, the heat of formation and chemical formula for liquid fraction were obtained experimentally. For gaseous fractions, the heat of formation data were obtained from NIST tables and the contributions of individual components were determined by GC calibrations. The value of x in equation 1 was experimentally determined to be 0.34. The overall hydrocarbon formula is slightly higher than C_2_H_4_ due to error in GC yields of gaseous components.


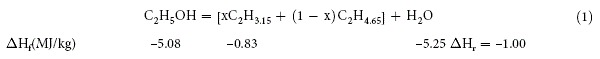


Since ethanol dehydration is endothermic (ΔHr 0.93 MJ/kg), the ethylene upgrading needs to be highly exothermic for overall reaction to be exothermic. The details of calculations are presented in Table S1 of supplementary section.

In order to determine if dehydration step in ethanol conversion is indeed the first step, we carried out experiments with deuterated ethanol, C_2_H_5_OD. We reasoned that deuterium will not get incorporated in the product stream if ethanol first dehydrates to ethylene or diethyl ether (Fig. S3). On the other hand, if ethanol conversion does not involve dehydration, product stream will contain deuterated hydrocarbons. Our results show that the conversion of C_2_H_5_OD or a mixture of 70% C_2_H_5_OH and 30% D_2_O results in deuterium incorporation in all hydrocarbons in the product stream including ethane except in ethylene. A likely explanation for deuterium incorporation is that hydrocarbon pool mechanism is a pathway for ethanol conversion to C_2+_ hydrocarbons and C_2_H_5_OD adds and eliminates across C=C containing organics (aliphatic or side chain on aromatics) inside zeolites resulting in deuterium incorporation.

The deuterium exchange of catalyst hydroxyl group as a pathway for deuterium incorporation in product stream could be easily ruled out since the product stream from catalytic conversion of a mixture of 70% C_2_H_4_ and 30% D_2_O did not show any deuterium incorporation. The cation in the catalyst also does not play a role in deuterium incorporation because, regardless of the cations (H or V) in the catalyst, the deuterium incorporation was seen in product streams when either C_2_H_5_OD or a mixture of 70% C_2_H_5_OH and 30% D_2_O are employed as reactants and no deuterium incorporation is observed in the product stream from a mixture of 70% C_2_H_4_ and 30% D_2_O.

The energy balance and deuteration experiments can be explained if we consider initiation, propagation, and termination steps of ethanol conversion individually. It is possible that initial hydrocarbon pool forms from ethylene via dehydration step but the formation of initial hydrocarbon pool from ethoxy groups cannot be ruled out. As such, we show both possibilities in Fig. S4. It is also quite challenging to determine if ethylene in the product stream is from dehydration or hydrocarbon pool. However, the conventional dehydration mechanism can be definitely ruled out based on deuterium labeling experiment. The propagation can occur via hydrocarbon pool pathway since free radical pathway does not allow for deuterium incorporation in the product stream. The hydrocarbon pool pathway allows for deuterium incorporation since ethanol can directly add to -C=C- in both olefin and aromatic cycles. After ethanol addition, the loss of water can occur with either deuterium or hydrogen on carbon adjacent to ethoxy group (Fig. S4). The termination of reaction occurs with gradual transformation of hydrocarbon pool into coke. This is supported by gradual increase in ethylene formation and decrease in C_3_ and higher hydrocarbon yields. The coking has been observed previously and decoking restores catalyst activity.

**Figure 4:**
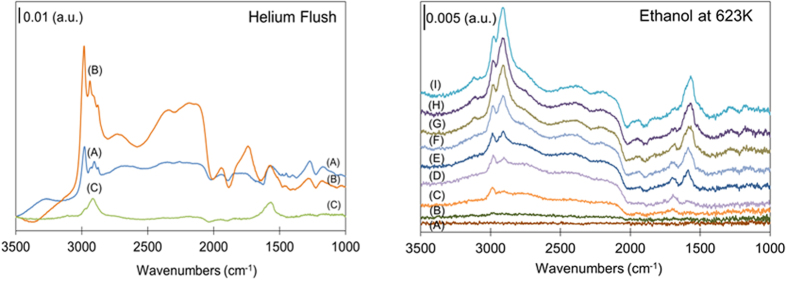
In-situ DRIFT absorbance spectra of samples exposed to ethanol and after flushing with helium for 10 minutes samples at (A) 25 °C, (B) 200 °C, and (C) 350 °C (**left**) and at 350 °C as a function of ethanol exposure time after (A) no exposure (B) 0.1 min; (C) 0.2 min; (D) 0.5 min; (E) 1.3 min; (F) 2.9 min; (G) 5.0 min; (H) 6.9 min; (I) 10.0 min (**right**).

The support for propagation via hydrocarbon pool pathway also comes from our diffuse reflectance infra-red studies into progress of ethanol upgrading over V-ZSM-5 (Fig. 4). Figure 4A compares the spectra after the samples had been flushed with helium for 10 min to clean the cell of gas phase products and unreacted ethanol. A low intensity, broad band around 3263 cm^−1^ was observed during the 25 °C study which can be attributed the O-H stretch of an alcohol. The absence of this band in either the 200 or 350 °C studies indicates that all ethanol adsorbed on the catalyst has reacted at these elevated temperatures which is consistent with experimental observation that all ethanol is converted to products at 200 °C or above. The C-H of the alkyl groups is present at all temperatures in the 2700–3000 cm^−1^ region and at ~1570 cm^−1^, respectively^57^. The presence of C-H and C=C stretches at 350 °C is consistent with experimental observation of formation of non-oxygenated hydrocarbon species. Our results match very well with those reported recently by Sousa *et al.*^58^ and essentially show that at temperature below 350 °C, ethoxy, diethyl ether, and ethylene species are present which are not observed above 350 °C.

We also carried out additional experiments to monitor changes in DRIFTS as a function of time after initial ethanol adsorption. A series of spectra, showing the changes that occur on the catalyst during the first 10 min, were captured immediately before and during the exposure of fresh V-ZSM-5 to ethanol flow at 350 °C (Fig. 4B). In the first 0.5 min of ethanol flow, before the C=C band of alkylated aromatics at 1567 cm^−1^ band appears, a band at 1690 cm^−1^ can be detected which may indicate the presence of linear alkenes. The band at 1567 cm^−1^ is not the dominant band in this region until ~1.3 min, after which the intensity of the band increases with time while that of 1690 cm^−1^ band decreases. The proposal of an initial alkene formation followed quickly by the formation of aromatics on the zeolite surface is further supported by the changes in the C-H stretching region. The absorption at 2987 cm^−1^ is the dominant C-H stretching band immediately after exposure to ethanol, while the 2911 cm^−1^ becomes dominant after roughly 3 min. The 2987 cm^−1^ does not diminish like 1690 cm^−1^ but increases at a slower rate than 2911 cm^−1^ and is red shifted from its original frequency to 2978 cm^−1^ 59,60. This may indicate that the position of the alkenes is no longer on the catalyst, but as substituents on the aromatics. These data suggest that linear alkene form at the initiation of reaction but aromatics become dominant within first minutes.

Thus, we have shown that heterobimetallic zeolite InV-ZSM-5 is more effective and durable in converting ethanol to liquid hydrocarbons. Our work with deuterium labeling does not conclusively prove any of the three proposed pathways in literature but supports hydrocarbon pool pathway and creates doubt about dehydration being the necessary first step.

## Methods

### Catalysts Synthesis

Commercial NH_4_-ZSM-5 (CBV2314) was purchased from Zeolyst Corporation. H-ZSM-5 was prepared by thermal treatment of commercial NH_4_-ZSM-5 at 500 °C for 4 h. V-ZSM-5 was prepared by a modification of a literature method^61^. NH_4_-ZSM-5 (SiO_2_/Al_2_O_3_ = 23) was soaked in V(III)Cl_3_ aqueous solution. Specifically, a 0.05 M solution of V(III)Cl_3_ was first made by dissolving 2.5 g of V(III)Cl_3_ into 320 mL of distilled water. Then, 12.17 g of NH_4_-ZSM-5 was added to the aqueous solution and warmed to 80 °C. After stirring for 16 hours under reflux condition, the mixture was vacuum filtered, and the solid was dried at 105 °C overnight. The light blue V-ZSM-5 solid was then calcined at 500 °C for four hours, which resulted in a light yellow final catalyst. In-ZSM-5 was synthesized using similar method as V-ZSM-5. Indium (III) nitrate was used as the precursor. For bimetallic InV-ZSM-5 preparation, V-ZSM-5 was first prepared with same V loading. Indium (III) nitrate aqueous solution of 0.015 M was prepared. V-ZSM-5 was then ion-changed with indium nitrate solution under reflux condition for 16 hours at 80 °C. After filtration, washing and drying at 105 °C, the catalyst was calcined at 500 °C for four hours with 1 °C/min ramping rate.

### Elemental Analysis

Elemental analyses were performed by Galbraith Incorporated, Knoxville, TN. V-ZSM-5 has about 0.7 wt% vanadium loading. Indium loading in In-ZSM-5 was 1.6 wt%. Bimetallic InV-ZSM-5 has 0.6 wt% vanadium and 1.6 wt% indium.

### UV-Vis-NIR and Raman analyses

Diffuse reflectance UV-Vis spectra were collected on a Cary 5000 UV-Vis-NIR spectrophotometer under R% mode. Raman spectroscopy was performed with an Alpha 300 confocal micro-Raman setup (WITec Inc., Germany) using a 532 nm excitation laser with a 20X objective and 600 g/mm (grooves per millimeter) grating. The laser power was attenuated to 300 uW and the laser spot size was approximately 1 um.

### *In-situ* diffuse reflectance FT infrared spectroscopy (DRIFTS) analysis

*In-situ* diffuse reflectance FT infrared spectroscopy (DRIFTS) measurements were performed on a Nicolet Nexus 670 spectrometer equipped with a MCT detector cooled by liquid nitrogen, and an *in-situ* chamber (HC-900, Pike Technologies) with capability to heat samples to 1173 K. The exiting stream was analyzed by an online quadrupole mass spectrometer (QMS) (OmniStar GSD-301 O2, Pfeiffer Vacuum). All samples studied by DRIFTS were heated to desired testing temperatures (room temperature (RT), 473 and 623 K) under a flow of 50 sccm of 100% helium and held at this temperature for at least 10 min before exposure to ethanol. A DRIFT spectrum of each fresh sample was taken at the desired testing temperature to confirm no IR active species were present before ethanol testing began. Ethanol reactant gas was created by injecting ethanol using a syringe pump at a rate of 0.04 mL/h into a heated flow of 50 sccm helium. Space velocity conditions were kept constant from sample to sample. A series of spectra were captured to monitor the changes in absorbance on the catalyst surface as a function of time upon exposure to the ethanol flow.

### Catalytic conversion of ethanol

A quartz reactor (8 mm ID × 25 cm H) was loaded with 0.2 g of catalyst (250–500 μm) between two layers of quartz wool. Two thermocouples were used to measure the temperatures, one in the middle of catalyst bed to measure catalyst temperature and the other one right below the quartz wool to measure the inlet gas temperature. All the reported temperatures in this paper are inlet gas temperature unless otherwise stated. The catalyst temperatures are similar for different catalysts at same inlet gas temperature. A tubular furnace was used to heat the catalyst up to 450 °C and hold for 0.5 h under 50 sccm dry helium flow. Then we changed it to catalyst testing temperatures and waited until the temperature stabilized. Pure ethanol (unless otherwise stated) was fed into the reactor employing a syringe pump. After stabilizing for 0.5 h, product analysis was performed by an on-line gas chromatograph (Agilent 6850) and mass spectrometer (5975C MS). The transfer line between the reactor and the GC/MS was heated to around 250 °C to prevent condensation of heavy products. Capillary column, HP-Plot Q, was used, with a dimension of 30.0 m × 320 μm × 20.0 μm. GC was held at 50 °C for 3 min, ramped up to 250 °C at 15 °C/min and then held for 35 min. Constant pressure mode of 9.51 psi was used and the inlet temperature was 250 °C. A gas calibration mixture (1% ethylene, 1% propene, 1% isobutene, 1% isopentane and balance nitrogen) was used to calibrate C_2_ to C_5_ hydrocarbons. Standards of benzene, toluene, p-xylene, ethylbenzene and cumene were used to quantify aromatic compounds. Nitrogen (5 sccm) was used as internal standard for all the analyses. For durability test, catalyst was regenerated under 20 ccm air flow with temperature ramped up to 450 °C at 2 °C/min, holding for 15 min, then ramping up to 500 °C and holding for 1 h.

### Liquid Hydrocarbon Analysis

The liquid product stream was collected in a liquid nitrogen trap. A sample of the liquid product stream was exposed to ambient temperature and pressure to allow evaporation of low-boiling products to allow for safe transportation. The sample was separated from water, dried over anhydrous MgSO_4_, and sent to SGS Oil, Gas, and Chemicals, Deer Park, TX 77536 for analysis (referred to as SGS analysis in the paper).

### Deuterium Labeling Experiments

A set of experiments involved employing deuterated ethanol. The rationale was that deuterium will not get incorporated if ethanol first dehydrates to ethylene or diethyl ether (Fig. S3). All these experiments were carried out in the quartz tube reactor (mentioned above). Products identification was performed using online GC/MS.

## Additional Information

**How to cite this article**: Narula, C. K. *et al.* Heterobimetallic Zeolite, InV-ZSM-5, Enables Efficient Conversion of Biomass Derived Ethanol to Renewable Hydrocarbons. *Sci. Rep.* **5**, 16039; doi: 10.1038/srep16039 (2015).

## Supplementary Material

Supplementary Information
