# Supplementary material for: Heterobimetallic Zeolite, InV-ZSM-5, Enables Efficient Conversion of Biomass Derived Ethanol to Renewable Hydrocarbons
Source: Sci Rep. 2015 Nov 3;5:16039. doi: 10.1038/srep16039 (PMC4630624; doi:10.1038/srep16039)
Supplement: Supplementary Information [file srep16039-s1.doc]

Supplementary Materials

HETEROBIMETALLIC ZEOLITE, InV-ZSM-5, ENABLES EFFICIENT CONERSION OF BIOMASS DERIVED ETHANOL TO RENEWABLE HYDROCARBONS

Chaitanya K. Narula,^1^* Zhenglong Li,^1^ Erik M. Casbeer,^1^ Robert A. Geiger,^1^ Melanie Moses-Debusk^2^, Martin Keller^2^, Michelle V. Buchanan^3^ & Brian H. Davison^2^

^1^Materials Science & Technology Division, Oak Ridge National Laboratory, Oak Ridge, TN, USA, 37831-6133

^2^Energy & Environmental Sciences Directorate, Oak Ridge National Laboratory, Oak Ridge, TN 37831

^3^Physical Sciences Directorate, Oak Ridge National Laboratory, Oak Ridge, TN 37831

*Correspondence to: [narulack@ornl.gov](mailto:narulack@ornl.gov)

**Table S1**. Heat of combustion calculation for ethanol conversion to hydrocarbons on V-ZSM-5

| Compound | ΔHf (MJ/kg) | Yield (%) | Contribution towards ΔHr (MJ/kg) |
| --- | --- | --- | --- |
| Ethanol | -5.08 | - | - |
| Blend-Stock (C5 and higher HC) | -2.07 | 20.2 | -0.42 |
| Ethylene | 1.87 | 6.8 | 0.13 |
| Ethane | -2.78 | 0.7 | -0.02 |
| Propane | -2.37 | 7.0 | -0.17 |
| Propene | 0.49 | 5.6 | 0.03 |
| *Iso*-butane | -2.31 | 12.2 | -0.28 |
| Butane | -2.16 | 4.1 | -0.09 |
| 2-Methyl-1-propene | -0.32 | 3.4 | -0.01 |
| 2-Butene | -0.16 | 0.9 | -0.001 |
| Water (g) | -13.44 | 39.1 | -5.25 |
| Total | - | 100 | -6.08 |

**Figure S1**. Ethanol conversion and oxygenates selectivity vs temperature for all the catalysts InV-ZSM-5, V-ZSM-5, In-ZSM-5 and H-ZSM-5 at WHSV of 1.6 h^-1^. Only one set of data is shown for these four catalysts as they are same.


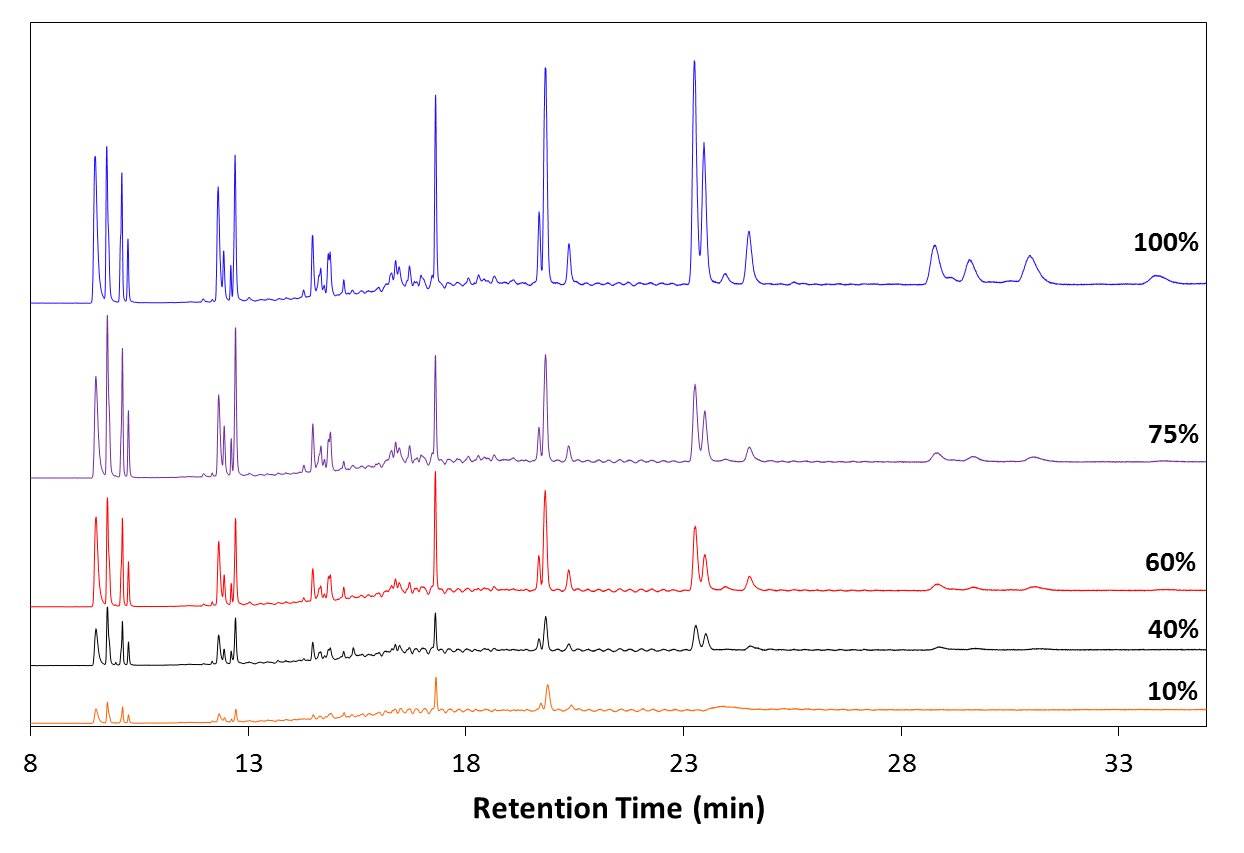


**Figure S2**. **Product distribution.** A comparison of gas chromatograms of hydrocarbon blend-stock product stream from the conversion of aqueous ethanol over V-ZSM-5 at 623K at WHSV of 1.6 h^-1^. The % on the right shows percentage of ethanol in aqueous ethanol solution.

**Figure S3**. **Ethanol-D dehydration pathways.** Pathways for ethanol-D dehydration, and mixture of ethanol and deuterated water dehydration.

**
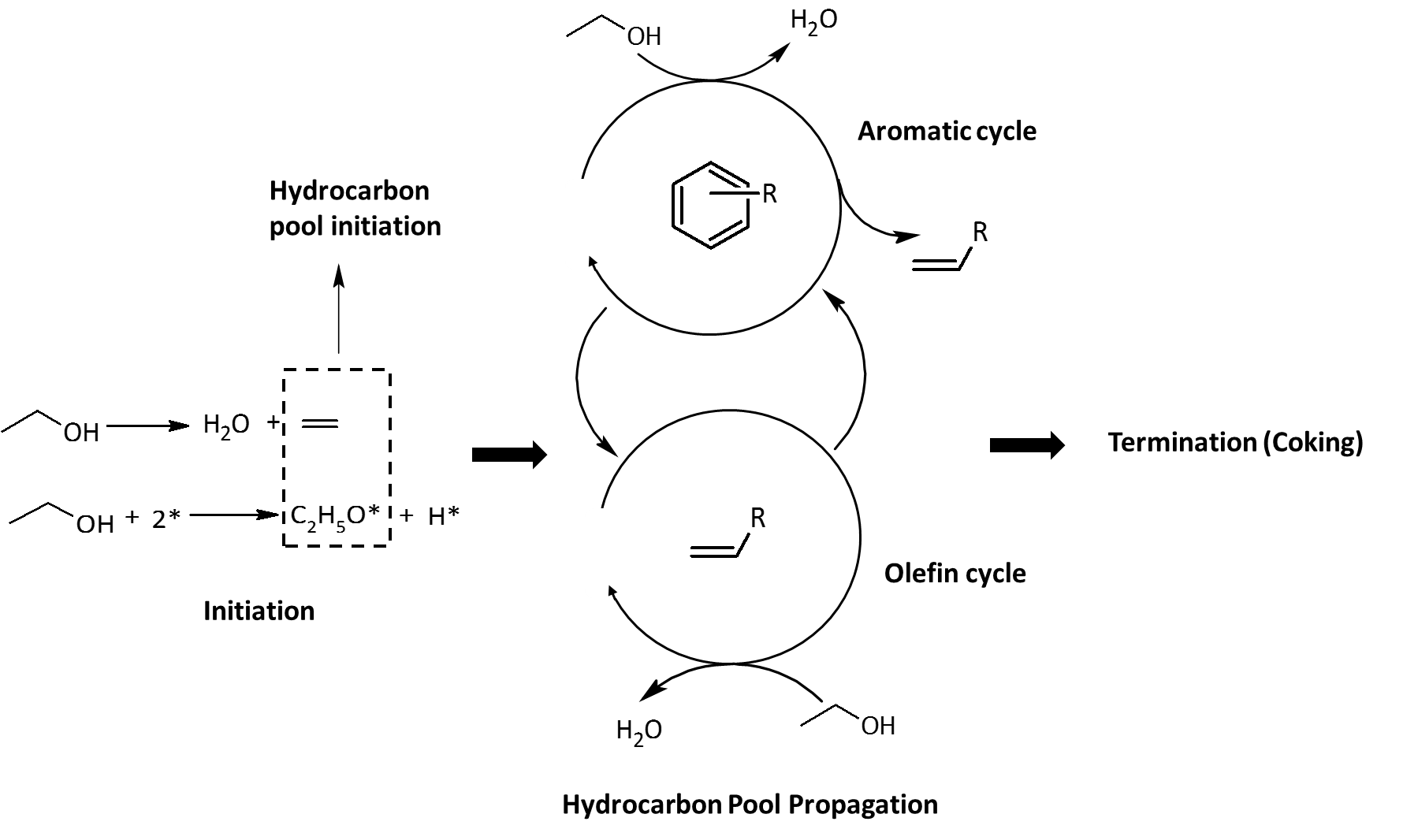
**

**Figure S4**. **Overall mechanism.** Proposed overall mechanism for ethanol conversion to hydrocarbon blend-stock. * is the adsorption site for ethoxy and H. The proposed mechanism is based on Methanol-to-Olefin conversion described in reference 39.
